# Publisher Correction: NDUFS4 regulates cristae remodeling in diabetic kidney disease

**DOI:** 10.1038/s41467-024-47414-1

**Published:** 2024-04-08

**Authors:** Koki Mise, Jianyin Long, Daniel L. Galvan, Zengchun Ye, Guizhen Fan, Rajesh Sharma, Irina I. Serysheva, Travis I. Moore, Collene R. Jeter, M. Anna Zal, Motoo Araki, Jun Wada, Paul T. Schumacker, Benny H. Chang, Farhad R. Danesh

**Affiliations:** 1 Section of Nephrology, The University of Texas MD Anderson Cancer Center, Houston, TX USA; 2 Department of Nephrology, Rheumatology, Endocrinology & Metabolism, Okayama University Graduate School of Medicine, Dentistry & Pharmaceutical Sciences, Okayama, Japan; 3 Department of Hematopathology, The University of Texas MD Anderson Cancer Center, Houston, TX USA; 4 Division of Nephrology, The Third Affiliated Hospital of Sun Yat-Sen University, Guangzhou, China; 5 Department of Biochemistry and Molecular Biology, The University of Texas Health Science Center at Houston, Houston, TX USA; 6 Department of Integrative Biology and Pharmacology, The University of Texas Health Science Center at Houston, Houston, TX USA; 7 Department of Epigenetics and Molecular Carcinogenesis, The University of Texas MD Anderson Cancer Center, Houston, TX USA; 8 Department of Urology, Okayama University Graduate School of Medicine, Dentistry & Pharmaceutical Sciences, Okayama, Japan; 9 Department of Pediatrics, Feinberg School of Medicine, Northwestern University, Chicago, IL USA; 10 Department of Biochemistry and Molecular Pharmacology, Baylor College of Medicine, Houston, TX USA

**Keywords:** Diabetic nephropathy, Mitochondria, Diabetes complications

Correction to: *Nature Communications* 10.1038/s41467-024-46366-w, published online 04 March 2024

In the original version of this Article, two asterisks were mistakenly introduced in Fig 1g. This has been corrected in both the PDF and HTML versions of the Article.

