# Supplementary material for: NDUFS4 regulates cristae remodeling in diabetic kidney disease
Source: Nat Commun. 2024 Mar 4;15:1965. doi: 10.1038/s41467-024-46366-w (PMC10912198; doi:10.1038/s41467-024-46366-w)
Supplement: Supplementary file 1 — Supplementary Information [file 41467_2024_46366_MOESM1_ESM.pdf]

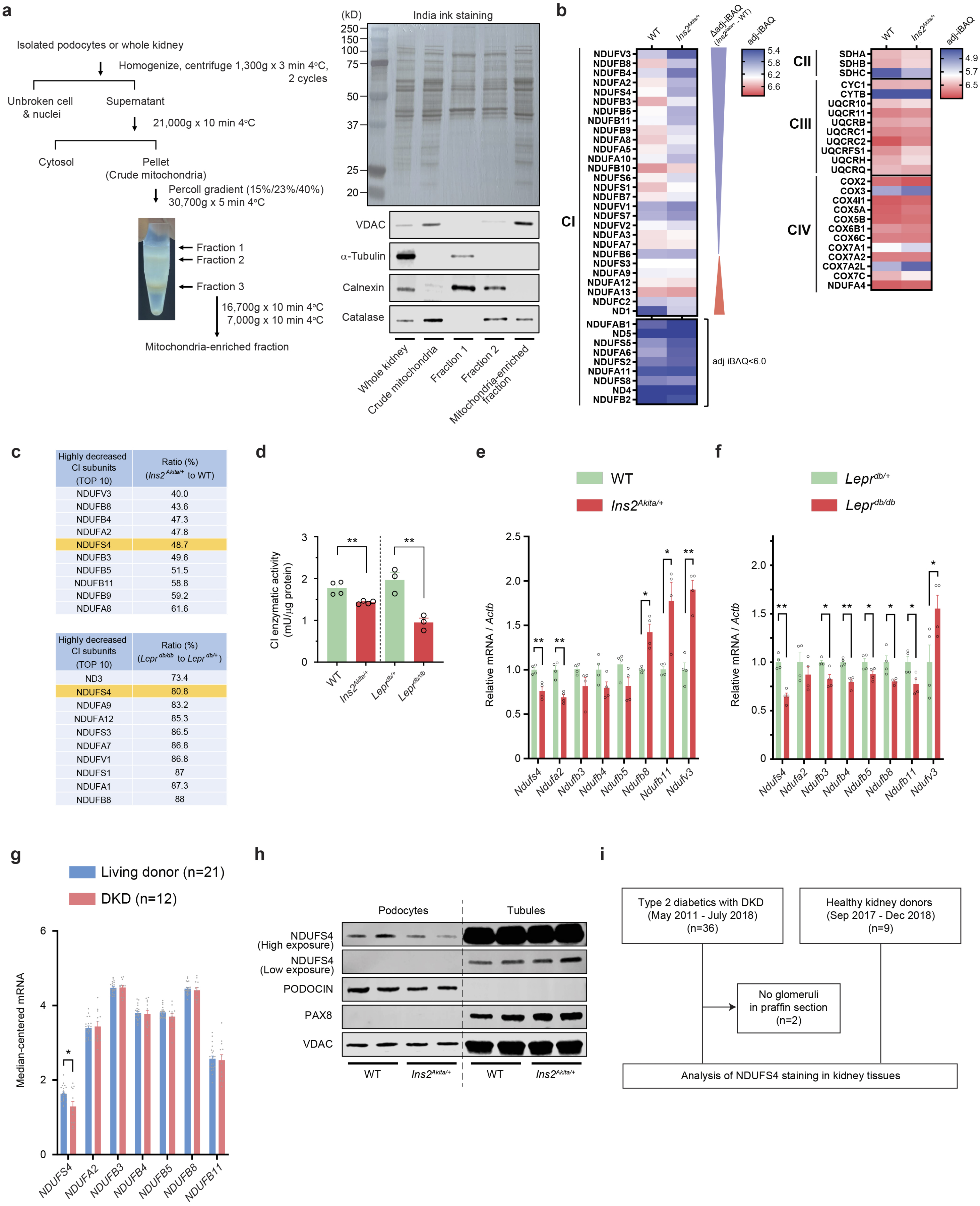

Highly decreased CI subunits (TOP 10)	Ratio (%) (*Ins2*$^{Akita/+}$ to WT)
NDUFV3	40.0
NDUFB8	43.6
NDUFB4	47.3
NDUFA2	47.8
NDUFS4	48.7
NDUFB3	49.6
NDUFB5	51.5
NDUFB11	58.8
NDUFB9	59.2
NDUFA8	61.6

Highly decreased CI subunits (TOP 10)	Ratio (%) (*Lepr*$^{db/db}$ to *Lepr*$^{db/+}$)
ND3	73.4
NDUFS4	80.8
NDUFA9	83.2
NDUFA12	85.3
NDUFS3	86.5
NDUFA7	86.8
NDUFV1	86.8
NDUFS1	87
NDUFA1	87.3
NDUFB8	88

Supplementary Fig. 1

Supplementary Fig. 1 | NDUFS4 expression is downregulated in podocytes, but not in tubules, in diabetic environment. a, Mitochondria isolation from podocytes or whole kidney using Percoll density gradient centrifugation (left). Immunoblots to validate the purity of mitochondria in different fractions (lower right). India ink staining was used to show all proteins (upper right). **b**, Proteomic profiling of mitochondrial proteins from primary podocytes of WT and *Ins2*$^{Akita/+}$ mice. Heatmaps of CI subunits (left panel), and CII-CIV subunits (right panel). **c**, Most down-regulated CI subunits in mitoproteomes of *Ins2*$^{Akita/+}$ and *Lepr*$^{db/db}$ diabetic mice compared to control mice. **d**, CI activity assessed by CI Enzyme Activity Microplate Assay Kit (n=4 (WT and *Ins2*$^{Akita/+}$), n=3 (*Lepr*$^{db/m}$ and *Lepr*$^{db/db}$)). **e,f,** qRT-PCR analysis of the relative mRNA expression of selective CI subunits (relative to *Actb*) in isolated podocytes from 16-week-old WT and *Ins2*$^{Akita/+}$ (**e**) and *Lepr*$^{db/+}$ *and Lepr*$^{db/db}$ mice (**f**) (n=4, each n represents a pool of RNA samples from 4 different mice). **g**, mRNA expression of selective CI subunits in the glomeruli of patients with DKD (Nephroseq v5) (Healthy living donor n=21, DKD patients n=12), median-centered Log_2 values are used for the analysis. **h**, Western blot analysis of NDUFS4 in primary podocytes and tubular cells isolated from WT and *Ins2*$^{Akita/+}$ mice, PODOCIN (podocyte marker), PAX8 (tubular marker), and VDAC (mitochondrial marker) were used as cell type specific markers. **i**, Study flowchart of the NDUFS4 staining analysis in kidney biopsies from DKD patients (n=34) and healthy donors (n=9). Data are mean ± SEM. **P* < 0.05, ***P* < 0.01, unpaired two-tailed *t* test, FDR Q< 0.05 (**e**-**g**). Source data are provided as a Source Data file.

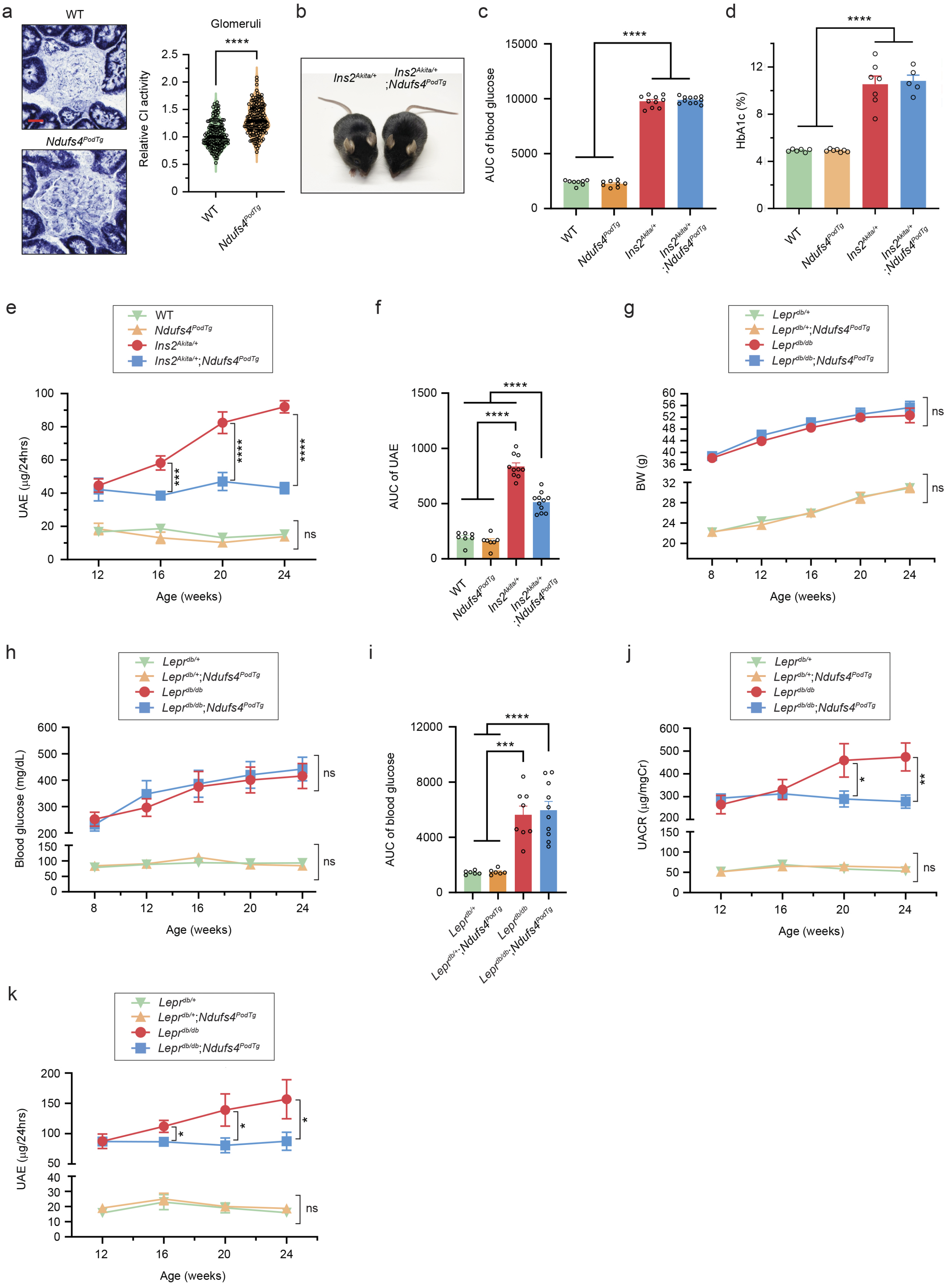

Supplementary Fig. 2

Supplemental Fig. 2 | Podocyte-specific Ndufs4 overexpression mitigates albuminuria in type 2 diabetic mice. a, Representative images of NADH oxidase activity in glomeruli from kidney sections of WT and *Ndufs4*^{PodTg} mice (left panel). Relative NADH oxidase activity in glomeruli (right panel) (n=150 from 3 mice/group). Scale bar=20 μm. **b**, Appearance of a diabetic (*Ins2*$^{Akita/+}$) and a diabetic *Ndufs4*^{PodTg} mouse at 18 weeks. **c**, Area under the curve (AUC) of blood glucose levels from 4-, 10-, 14-, 18- and 23-week-old mice shown in Fig. 2f (n=8 (WT and *Ndufs4*^{PodTg}), n=10 (*Ins2*$^{Akita/+}$), n=11 (*Ins2*$^{Akita/+}$;*Ndufs4*^{PodTg})). **d**, Hemoglobin A1C (HbA1c) levels in four different groups of mice (n=6 (WT), n=7 (*Ndufs4*^{PodTg}), n=7 (*Ins2*$^{Akita/+}$), n=5 (*Ins2*$^{Akita/+}$;*Ndufs4*^{PodTg})). **e**, Urinary albumin excretion (UAE, μg/24hrs) in 12-, 16-, 20- and 24-week-old mice. **f**, AUC of UAE shown in Supplementary Fig. 2e (e,f n=7 (WT and *Ndufs4*^{PodTg}), n=10 (*Ins2*$^{Akita/+}$), n=11 (*Ins2*$^{Akita/+}$;*Ndufs4*^{PodTg})). **g**-**k**, Body weight (g, n=7 (*Lepr*$^{db/m}$ and *Lepr*$^{db/m}$;*Ndufs4*^{PodTg}), n=9 (*Lepr*$^{db/db}$ and *Lepr*$^{db/db}$;*Ndufs4*^{PodTg})), blood glucose (**h**), blood glucose AUC (**i**) (h,i, n=6 (*Lepr*$^{db/m}$ and *Lepr*$^{db/m}$-*Ndufs4*^{PodTg}), n=8 (*Lepr*$^{db/db}$), n=10 (*Lepr*$^{db/db}$;*Ndufs4*^{PodTg})), urinary albumin-to-creatinine ratio (UACR) (**j**), and UAE (**k**) of control and type 2 diabetic *Lepr*$^{db/db}$ mice at different ages (j, k, n=7 (*Lepr*$^{db/m}$, *Lepr*$^{db/m}$;*Ndufs4*^{PodTg}, and *Lepr*$^{db/db}$), n=8 (*Lepr*$^{db/db}$;*Ndufs4*^{PodTg})). Results are presented as median ± IQR (**a**, bold line: median, and dot line: IQR) or mean ± SEM (**c**-**k**). ns, not significant *$P < 0.05$, ** $P < 0.01$, *** $P < 0.001$, **** $P < 0.0001$. Mann-Whitney test (**a**), One-way ANOVA with post-hoc Tukey-Kramer test (**c**-**k**). Source data are provided as a Source Data file.

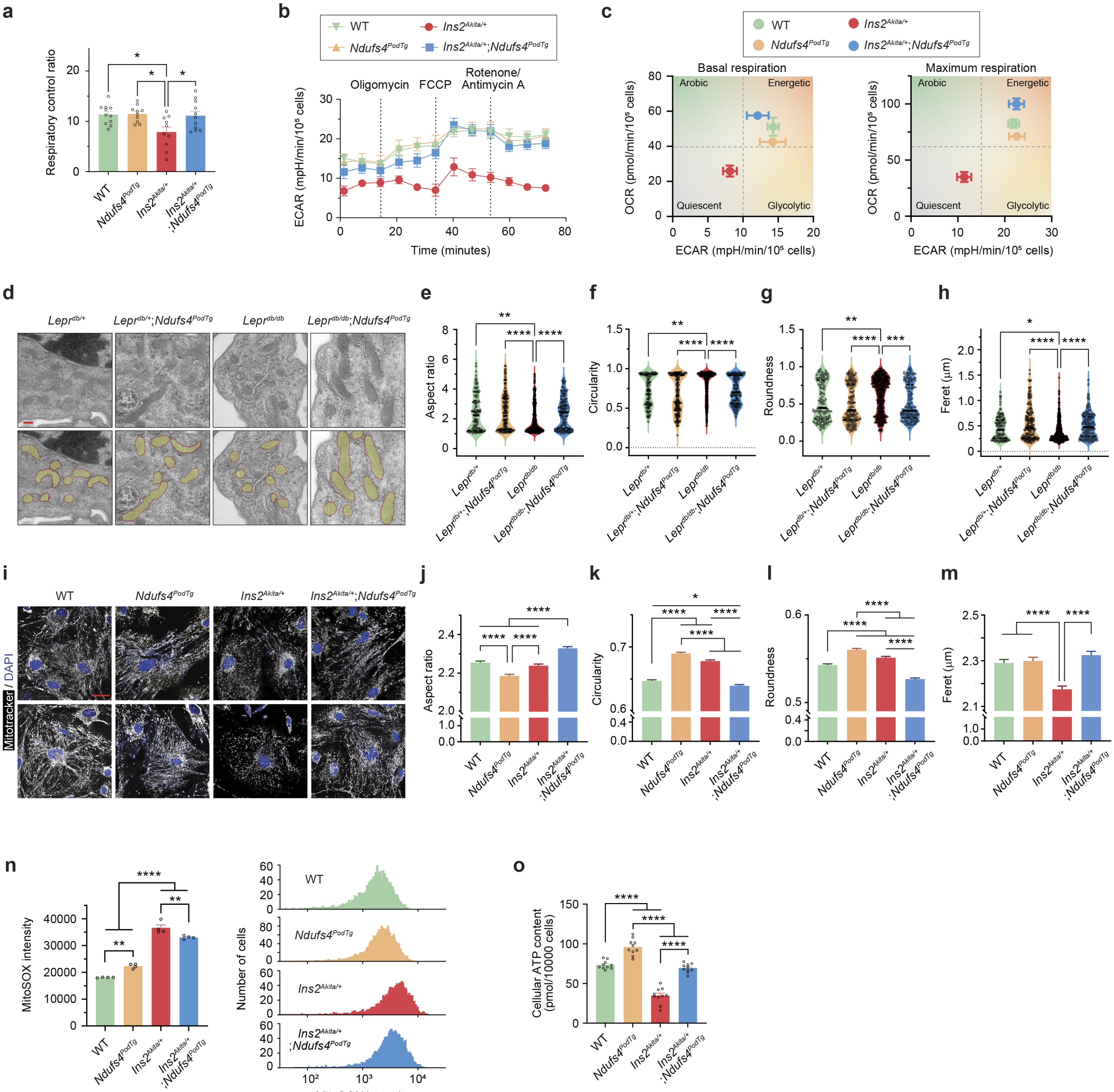

Supplementary Fig. 3

Supplementary Fig. 3 | Ndufs4 overexpression prevents mitochondrial fission in podocytes from type1 and type 2 diabetic mice. a,**b**, Seahorse analysis of respiratory control ratio (RCR; **a**) and extracellular acidification rate (ECAR; **b**) using primary podocytes isolated from WT, $Ndufs4^{PodTg}$, $Ins2^{Akita/+}$, and $Ins2^{Akita/+}$;$Ndufs4^{PodTg}$ mice (a, n=11 (WT and $Ndufs4^{PodTg}$;$Ins2^{Akita/+}$), n=10 ($Ndufs4^{PodTg}$ and $Ins2^{Akita/+}$), replicates/group, b, n=6 (WT), n=7 ($Ins2^{Akita/+}$ and $Ndufs4^{PodTg}$;$Ins2^{Akita/+}$), n=8 ($Ndufs4^{PodTg}$), replicates/group). **c**, Energy maps displaying the OCR and the EACR under basal and maximal respirations in primary podocytes isolated from four experimental groups of mice. **d-h**, Representative TEM micrographs of podocyte mitochondria from kidney tissues (**d**, top) and pseudo-color superimposed images (**d**, bottom) to assess mitochondrial morphological changes in aspect ratio (**e**), circularity (**f**), roundness (**g**), and feret diameter (**h**) (n=115-454 from 3 mice/group) Scale bar=200 nm. **i**-**m**, Representative immunofluorescent images (**i**) of primary podocytes stained with mitotracker (white) showing mitochondrial morphological changes in aspect ratio (**j**), circularity (**k**), roundness (**l**), and feret diameter (**m**) (n=21675-23513/group) Scale bar=40 μm. **n,** Median intensity of MitoSOX Red staining (left panel, n=4 replicates/group), distribution of cells sorted by MitoSOX intensity (right panel, n=6572-11739 cells/group). **o,** Cellular ATP content (n=10 replicates/group). Data are presented as median ± IQR (**e**-**h**, bold line: median, and dot line: IQR) or mean ± SEM (**a,b,c, j**-**o**) *P < 0.05, **P < 0.01, ***P < 0.001, ****P < 0.0001. Kruskal-Wallis with post-hoc Dunn's test (**e**-**h**) and One-way ANOVA with post-hoc Tukey-Kramer test (**a,j**-**o**). Source data are provided as a Source Data file.

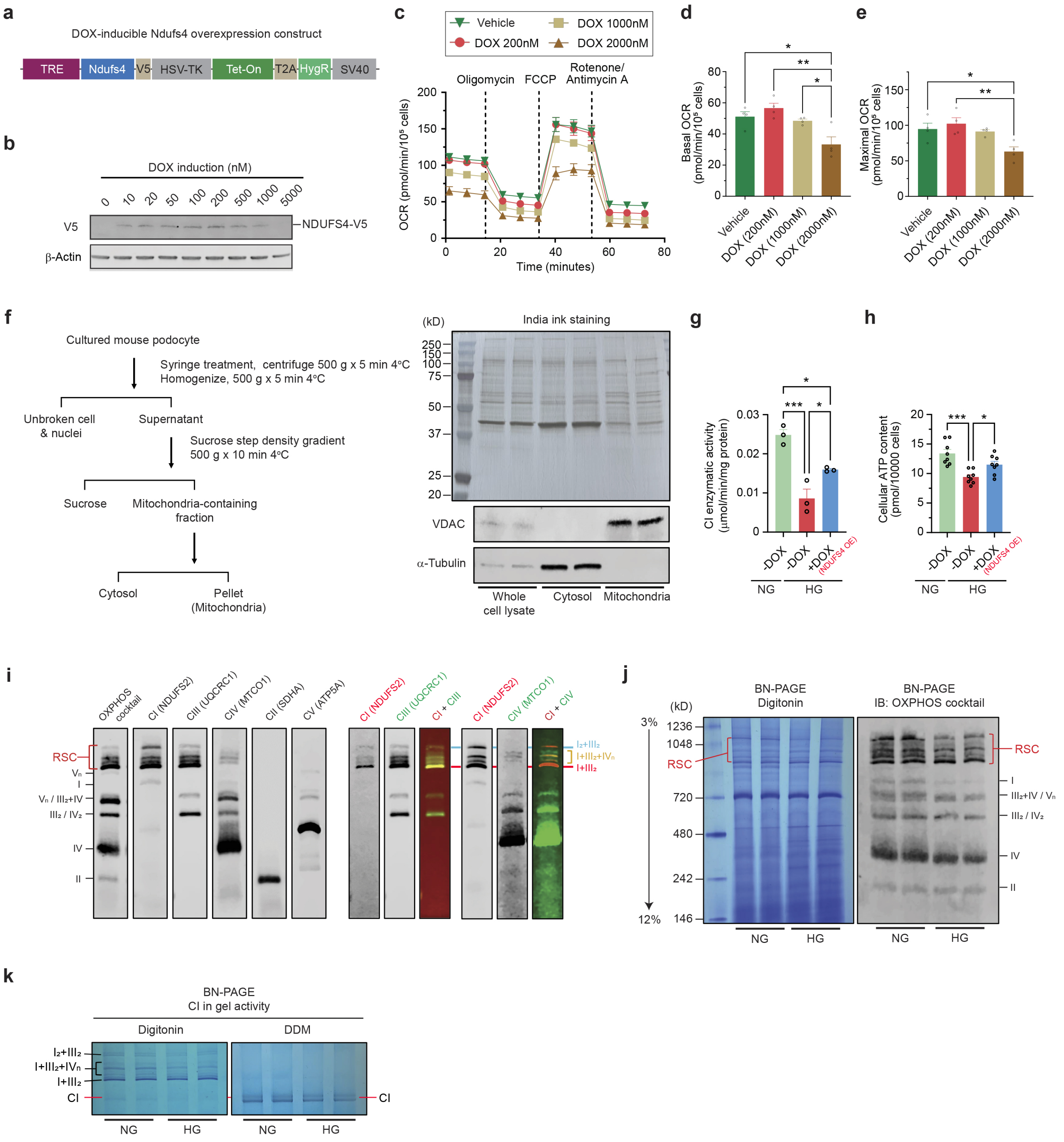

Supplementary Fig. 4

Supplementary Fig. 4 | DOX-induced NDUFS4 OE in podocytes improves HG-induced mitochondrial remodeling. **a**, *PiggyBac* transposon vector containing a doxycycline (DOX)-inducible Ndufs4 (NDUFS4 OE) expression cassette. **b**, NDUFS4 expression induced by DOX at different concentrations. **c,** OCRs in podocytes (n=4 replicates/group) treated with control or different dose of DOX. Dotted lines denote 3 injections of oligomycin (2 μM), FCCP (2 μM), rotenone, and antimycin A (both 0.5 μM). **d,e,** Basal respiration (**d**) and maximal respiration (**e**) derived from Supplementary Fig. 4c. **f,** Procedure of mitochondrial isolation by sucrose step density gradient centrifugation using cultured podocytes (left panel), and immunoblot validation of mitochondrial purity in cytosol, and mitochondrial-enriched fraction (bottom right panel). Protein extracts from whole cell lysate was used as a control. India ink staining (upper right panel) showed the amounts of proteins loaded. **g**,**h**, Rotenone-sensitive CI enzymatic activity in isolated mitochondria (g**,** n=3/group) and ATP content (h**,** n=8 replicates/group) in podocytes cultured under NG (5.5 mM) and HG (25 mM) for 48hrs with (NDUFS4 OE) or without DOX induction. **i**, Immunoblots of BN-PAGE of digitonin-solubilized mitochondria from DOX-induced NDUFS4 OE podocytes showing individual complexes as well as stoichiometry of respiratory supercomplexes (RSC). **j**, Coomassie staining (left panel) and immunoblot (right panel) of BN-PAGE analysis of digitonin-solubilized mitochondria isolated from podocytes cultured under NG and HG for 48hrs. **k**, CI in-gel activity of digitonin- and n-Dodecyl β-D-maltoside (DDM)-solubilized mitochondria from the same cells as in Supplementary Fig. 4j. RSC: respirasome supercomplexes. Roman numerals indicate RSC with defined stoichiometry of individual complexes. Data are presented as mean ± SEM (**c**-**e**,**g**,**h**) **P* < 0.05, ****P* < 0.001, *****P* < 0.0001. One-way ANOVA with post-hoc Tukey-Kramer test (**d**,**e**,**g**,**h**). Source data are provided as a Source Data file.

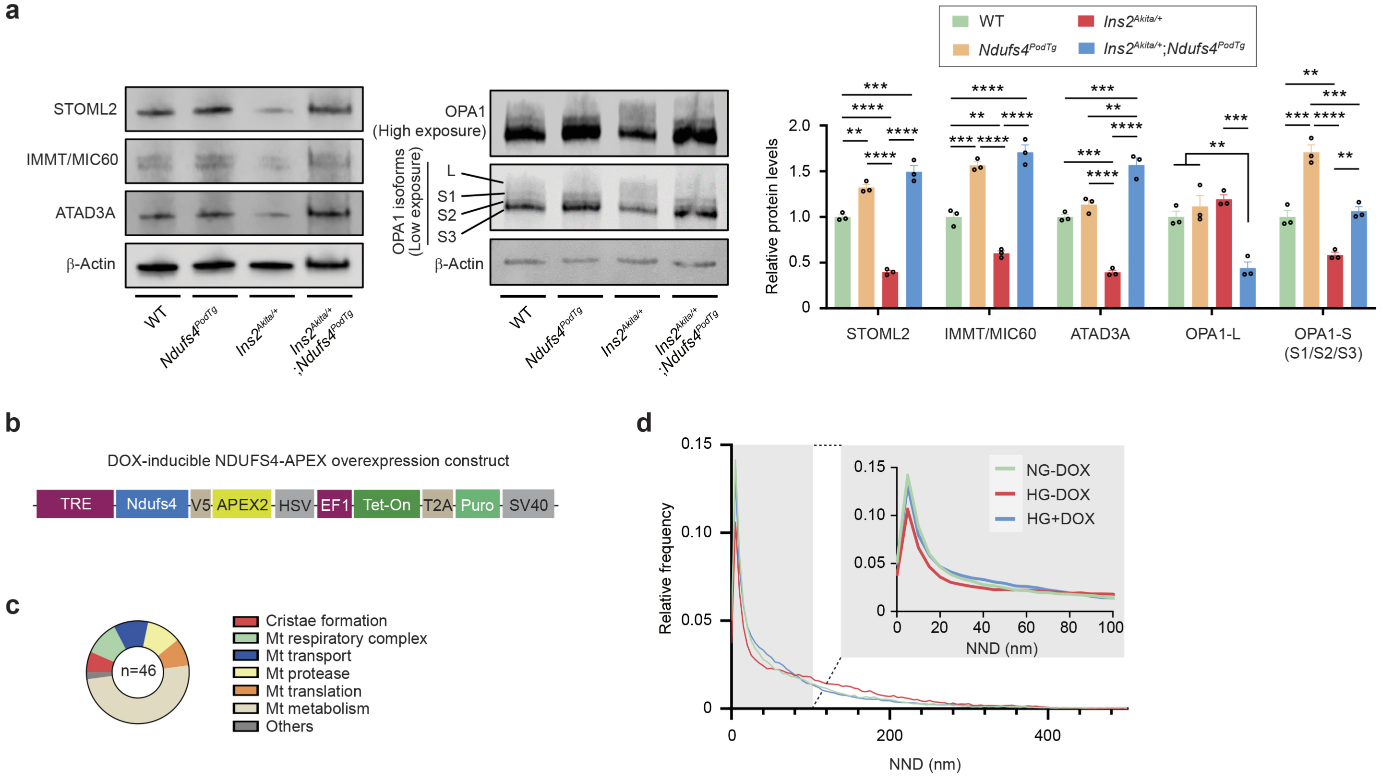

Supplementary Fig. 5

Supplementary Fig. 5 | Doxycycline-inducible Ndufs4-APEX construct and NDUFS4-interactome. a, Immunoblotting of STOML2, IMMT/MIC60, ATAD3, and OPA1 in primary podocytes (left panel) with OPA1 isoforms in detail analysis (middle panel), and quantitative analysis (right panel) (n=3). β-Actin was used for loading control. **b**, A schematic depiction of the *PiggyBac* transposon vector containing a doxycycline (DOX)-inducible NDUFS4-APEX expression cassette. **c**, The top 46 NDUFS4 associated mitochondrial proteins categorized into various groups according to their biological functions. Mt: mitochondrial. **d**, Trajectory of histograms for nearest neighboring distance (NND). Histograms show the distribution of the distance between NDUFS4 molecule and nearest neighboring STOML2 molecule. Data are presented as mean ± SEM (**a**) $^{**}P < 0.01$, $^{***}P < 0.001$, $^{****}P < 0.0001$. One-way ANOVA with post-hoc Tukey-Kramer test (**a**). Source data are provided as a Source Data file.

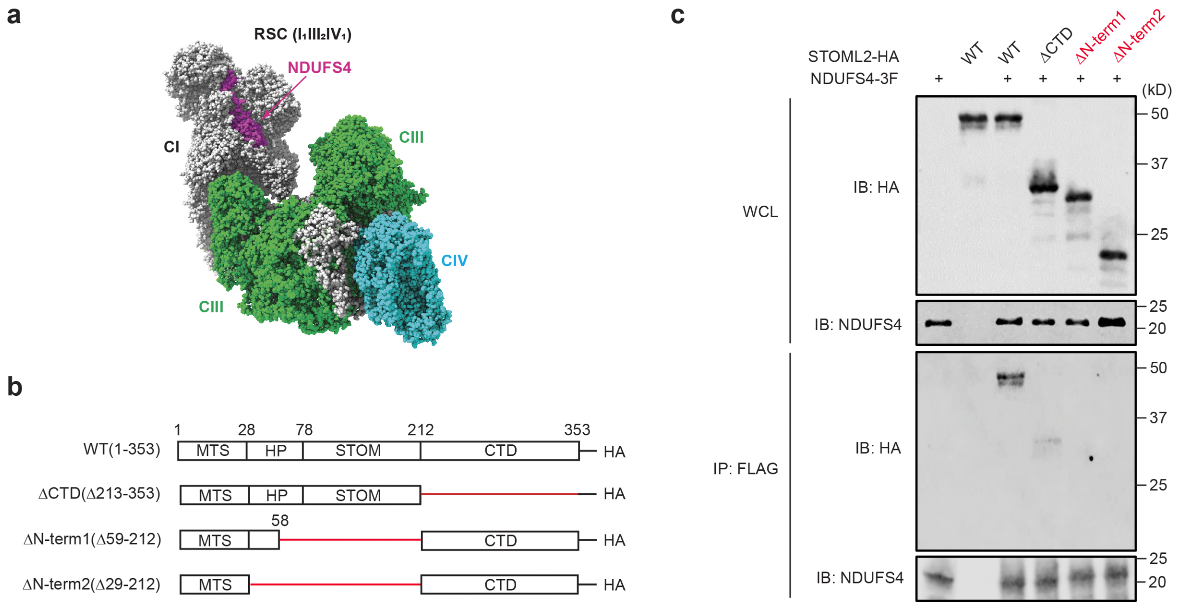

Supplementary Fig. 6

Supplementary Fig. 6 | STOML2 binds NDUFS4 at N-terminus domain. a, Structure of RSC ($I_1III_2IV_1$; PDB-5XTH) visualized with UCSF ChimeraX (https://www.cgl.ucsf.edu/chimerax/). CI (gray), CIII (green), CIV (Cyan), and NDUFS4 (magenta) are highlighted by pseudo-coloring. **b**, Structure of mouse STOML2 WT and deletion mutant constructs engineered with HA-tag at the C-terminus. MTS: mitochondrial targeting sequence. HP: hydrophobic hairpin. STOM: stomatin. CTD: C-terminal coiled-coil domain. ∆CTD: deletion mutant of c-terminus mutant (∆213-353). ∆N-term1: deletion mutant of N-terminus 1 (∆59-212). ∆N-term2: deletion mutant of HP and STOM (∆29-212). **c**, Co-IP assay of NDUFS4 and STOML2 in HEK293T cells transfected with NDUFS4-FLAG and indicated STOML2-HA constructs shown in Supplementary Fig. 6b. FLAG antibody-conjugated beads were used for Co-IP and anti-HA or anti-NDUFS4 antibodies were used for immunoblots. WCL: whole cell lysate. Source data are provided as a Source Data file.

Supplementary Table 1. Clinical parameters of donors and DKD patients at renal biopsy.

Clinical parameters	Donor (n=9)	DKD (n=34)	*P*-value
Male (%)	44	59	0.477
Age (years)	56 ± 13	57 ± 12	0.933
BMI (kg/m^2)	20.9 ± 2.5	25.6 ± 5.4	0.015
Duration of DM (years)*	-	5.5 (2.0-15.0)	-
HbA1c (%)	5.7 ± 0.2	7.2 ± 1.6	0.009
SBP (mmHg)	115.8 ± 12.6	146.0 ± 21.7	0.0003
DBP (mmHg)	70.4 ± 12.9	83.1 ± 9.5	0.002
MAP (mmHg)	85.6 ± 10.5	104.1 ± 11.7	0.0001
Hypertension (%)†	33	85	0.004
Retinopathy (%)	-	62	-
sCr (mg/dl)*	0.71 (0.60-0.84)	1.1 (0.91-1.78)	0.002
eGFR (ml/min/1.73m^2)	79.0 ± 7.1	50.6 ± 25.8	0.002
CKD GFR Categories (G1/G2/G3/G4/G5, %)	11/89/0/0/0	6/35/32/18/9	0.037
UACR (mg/gCr)*	6.6 (4.3-13.0)	1675 (629-3030)	0.0001
Normo/Micro/Macro-1/Macro-2 (%)	100/0/0/0	12/6/26/56	<0.0001

Abbreviations; BMI, body mass index; Duration of DM, estimated duration of diabetes mellitus; SBP, systolic blood pressure; DBP, diastolic blood pressure; MAP, mean arterial pressure; Retinopathy, diabetic retinopathy; sCr, serum creatinine; eGFR, estimated glomerular filtration rate; CKD GFR Categories G1 ≥90 ml/min/1.73m^2, G2 60-90 ml/min/1.73m^2, G3 30-59 ml/min/1.73m^2, G4 15-29 ml/min/1.73m^2, G5 <15 ml/min/1.73m^2; UACR, urinary albumin creatinine ratio; Normo/Micro/Macro: normoalbuminuria, microalbuminuria and macroalbuminuria. Macroalbuminuric patients were divided into: Macro-1: >300 but ≤1000mg/gCr of albuminuria and Macro-2: >1000mg/gCr of albuminuria. *Median (interquartile range). †Hypertension was defined as blood pressure ≥ 140/90 mmHg or the use of antihypertensive drugs.
Comparisons between two groups were performed using two-tailed Student's *t*-test for normally distributed data and two-tailed Mann-Whitney test for non-normally distributed data. Categorical variables were compared with the Fisher exact test.

Supplementary Table 2. List of primer sequences used for qRT-PCR analysis in this study.

Gene	Forward Primer (5'-3')	Reverse Primer (5'-3')
Actb	CTAAGGCCAACCGTGAAAAG	ACCAGAGGCATACAGGGACA
Hprt	AGGGATTTGAATCACGTTTG	TTTACTGGCAACATCAACAG
Ndufs4	GTCTGTAGAGTTCCATCCAG	GAGCAGGAACAAAGATTCTG
Ndufa2	GAACAATCTGAGTGCTGATG	CGTAAGCTTTATAAGGACCC
Ndufb3	GGTAGCTTTGGGGGCTGAAT	GTCACAAGGCGCTCTCTTCA
Ndufb4	TTTTCAAAACAGACAGGGAC	ATCCTTGCCGAACTTAGTAG
Ndufb5	GTCGTCAAGCCTTCTTTATAC	TCGGATGCTTGTAATACTCC
Ndufb8	CATGTGTAAACATCTCTTCGG	TCCTCAGATATCATAGTGAACC
Ndufb11	CTCAAAAACAACTCTCTCCC	AAGTTTGTTTACGAGTTCGG
Ndufv3	AAAGTGTGCTCAAAGATGTG	TTTCTTGACAAATGCTTCGG

Supplementary Table 3. List of antibodies and dyes used in this study.

Primary antibodies	Source	Catalog number
Anti-GST Alexa Fluor 680	Santa Cruz	sc-138 AF680
Goat anti-Podocalyxin, biotinylated	R&D Systems	BAF1556
Guinea pig anti-Synaptopodin	Progen	GP94-N
Mouse anti-β-Actin	Cell Signaling	4967
Mouse anti–FLAG M2 monoclonal	Sigma-Aldrich	F3165
Mouse anti-STOML2	Proteintech	60052-1-Ig
Mouse anti-VDAC	Abcam	ab14734
Mouse OXPHOS cocktail	Thermo Fisher	45-8099
Rabbit anti-ATAD3A/B	Proteintech	16610-1-AP
Rabbit anti-α-Tubulin	Cell Signaling	2144
Rabbit anti-Calnexin	Proteintech	10427-2-AP
Rabbit anti-Catalase	Cell Signaling	14097
Rabbit anti-HA-Tag	Cell Signaling	3724
Rabbit anti-Mitofilin	Proteintech	10179-1-AP
Rabbit anti-Ndufs4	Nobus	NBP1-31465
Rabbit anti-Ndufs4	Abcam	ab137064
Rabbit anti-OPA1	BD Biosciences	612606
Rabbit anti-PAX8	Proteintech	10336-1-AP
Rabbit anti-Podocin	Sigma-Aldrich	P0372
Rabbit anti-STOML2	Proteintech	10348-1-AP
Rabbit anti-VDAC	Cell Signaling	4661
Rabbit anti-V5-Tag	Cell Signaling	13202
Rabbit anti-Wilms Tumor Protein	Abcam	ab89901
Sheep anti-KIRREL3, biotinylated	R&D Systems	BAF4910

Secondary antibodies	Source	Catalog number
Donkey anti–mouse Alexa Fluor 488	Thermo Fisher	A21202
Donkey anti–mouse Alexa Fluor 594	Thermo Fisher	A21203
Donkey anti–rabbit Alexa Fluor 488	Thermo Fisher	A21206
Donkey anti–rabbit Alexa Fluor 594	Thermo Fisher	A21207
Donkey anti–rabbit Alexa Fluor 647	Thermo Fisher	A21244
Goat anti-guinea pig Alexa Fluor 594	Thermo Fisher	A11076
Goat anti–mouse DyLight 680	Thermo Fisher	35519
Goat anti–mouse DyLight 800	Thermo Fisher	SA510172
Goat anti-mouse ATTO 488	Rockland	610-152-121
Goat anti–rabbit DyLight 680	Thermo Fisher	35568
Goat anti–rabbit DyLight 800	Thermo Fisher	SA535571
Goat anti–rabbit IgG HRP Polymer	Vector Laboratories	MP-7451

Dyes	Source	Catalog number

DAPI	Thermo Fisher	62248
Mitotracker Red	Thermo Fisher	M7512
