# Supplementary material for: NDUFS4 regulates cristae remodeling in diabetic kidney disease
Source: Nat Commun. 2024 Mar 4;15:1965. doi: 10.1038/s41467-024-46366-w (PMC10912198; doi:10.1038/s41467-024-46366-w)
Supplement: Supplementary file 2 — Reporting Summary [file 41467_2024_46366_MOESM2_ESM.pdf]

nature portfolio

Corresponding author(s): Farhad R. Danesh

Last updated by author(s): Jan 12, 2024

Reporting Summary

Nature Portfolio wishes to improve the reproducibility of the work that we publish. This form provides structure for consistency and transparency in reporting. For further information on Nature Portfolio policies, see our Editorial Policies and the Editorial Policy Checklist.

Statistics

For all statistical analyses, confirm that the following items are present in the figure legend, table legend, main text, or Methods section.

n/a	Confirmed	
☐	☒	The exact sample size (*n*) for each experimental group/condition, given as a discrete number and unit of measurement
☐	☒	A statement on whether measurements were taken from distinct samples or whether the same sample was measured repeatedly
☐	☒	The statistical test(s) used AND whether they are one- or two-sided<br>*Only common tests should be described solely by name; describe more complex techniques in the Methods section.*
☒	☐	A description of all covariates tested
☐	☒	A description of any assumptions or corrections, such as tests of normality and adjustment for multiple comparisons
☐	☒	A full description of the statistical parameters including central tendency (e.g. means) or other basic estimates (e.g. regression coefficient) AND variation (e.g. standard deviation) or associated estimates of uncertainty (e.g. confidence intervals)
☐	☒	For null hypothesis testing, the test statistic (e.g. *F*, *t*, *r*) with confidence intervals, effect sizes, degrees of freedom and *P* value noted<br>*Give P values as exact values whenever suitable.*
☒	☐	For Bayesian analysis, information on the choice of priors and Markov chain Monte Carlo settings
☒	☐	For hierarchical and complex designs, identification of the appropriate level for tests and full reporting of outcomes
☐	☒	Estimates of effect sizes (e.g. Cohen's *d*, Pearson's *r*), indicating how they were calculated

Our web collection on statistics for biologists contains articles on many of the points above.

Software and code

Policy information about availability of computer code

Data collection	LC-MS/MS analysis was carried out using a nano-LC 1200 system coupled to Orbitrap Lumos ETD mass spectrometer (Thermo Fisher). Respiration analysis was performed on a Seahorse XFe96 analyzer. Flow cytometry was performed using FACS Aria (Becton Dickinson, San Jose, CA). Odyssey Fc Imaging System was used to obtain western blot images. StepOnePlus Real-Time PCR System was used to collect RT-qPCR data. Immunofluorescence images except for STED/STORM were captured by FV1200 MPE confocal microscope (Olympus). N-STORM (Nikon Instruments) was used for STORM imaging, and STED imaging was performed on an STEDYCON (Abberior) and Eclipse Ti2 inverted imaging system (Nikon Instruments) with a Plan Apochromat 100X (NA 1.49) oil immersion objective (Nikon Instruments). Cryo-ET data collection was performed using a Titan Krios G3 300 keV FEG transmission electron cryo-microscope (Thermo Fisher). Molecular graphics and visualization in Cryo-ET were performed with UCSF Chimera. Human STOML2 structure was predicted by Contact-guided Iterative Threading ASSEmbly Refinement web tool (C-I-TASSER). Human NDUFS4 within the structure of the respirasome was obtained from the PDB database (5XTB). For assessment of mitochondrial roGFP in podocytes, primary podocytes transduced with lenti-mito-roGFP were imaged by laser scanning confocal microscopy using a Leica SP8 confocal with a 40X oil (1.3 N.A.) Plan/Apo objective using 2 excitations, 405 nm and 488 nm to preferentially excite oxidized roGFP and reduced roGFP, respectively. Emitted light from both excitations were collected on the same HyD detector (498-551 nm) with pinhole aperture 1.3.
Data analysis	Image Studio (Li-COR, v5.2.5) was used for densitometry analysis for western blot. NDUFS4 intensity and NADH oxidase staining in glomeruli, and mitochondrial morphology were analyzed in Image J (Fiji, v2.0.0). The MS raw data was searched using Proteome Discoverer 2.1 software (Thermo Fisher) with Mascot algorithm against mouse NCBI refseq database updated 2020_0324. In STED analysis, JACoP plugin of mage J was used, and for STORM imaging analysis, ThunderSTORM plugin of image J was used. Maximum intensity projection (MIP) images were reconstructed from Nikon Nd2 files using Huygens Essential (Scientific Volume Imaging). SerialEM software was used for imaging of Cryo-ET. All statistical analyses were performed with GraphPad Prism version 9.3.1 (Graphpad Software). The ClusPro 2.0 web server25 was used to perform protein-protein docking simulation. The resulting docking structures were analyzed using PyMol Molecular Graphics Systems, version

2.0, Schrodinger, LLC. Images for mito-roGFP were quantitatively analyzed using Imaris (Andor/Oxford Instruments, version 9.9) to determine mitochondrial redox ratio according to the mean fluorescence intensity (MFI) per channel.

For manuscripts utilizing custom algorithms or software that are central to the research but not yet described in published literature, software must be made available to editors and reviewers. We strongly encourage code deposition in a community repository (e.g. GitHub). See the Nature Portfolio guidelines for submitting code & software for further information.

Data

Policy information about availability of data

All manuscripts must include a data availability statement. This statement should provide the following information, where applicable:

- Accession codes, unique identifiers, or web links for publicly available datasets
- A description of any restrictions on data availability
- For clinical datasets or third party data, please ensure that the statement adheres to our policy

All data supporting the findings of this study are available in the main text or supplementary materials. The mass spectrometry proteomic data for mitochondrial proteome in murine podocytes, NDUFS4-APEX proteome, and murine podocyte complexsome have been deposited to the ProteomeXchange Consortium via the PRIDE partner repository with the dataset identifiers, PXD041202, PXD045828, PXD041378, and PXD041203. Human NDUFS4 within the structure of the respirasome and the structure of the I1III2IV1 within RSC were obtained from the PDB database 5XTB and 5XTH, respectively. Glomerular transcriptomic data (Nephroseq database version 5) were analyzed using Ju CKD Glom dataset (GSE104948).

Research involving human participants, their data, or biological material

Policy information about studies with human participants or human data. See also policy information about sex, gender (identity/presentation), and sexual orientation and race, ethnicity and racism.

Reporting on sex and gender

Male and Female.

Reporting on race, ethnicity, or other socially relevant groupings

Japanese

Population characteristics

1. Patients with biopsy-proven diabetic kidney disease
age: 20-80 years when obtaining informed consent.
Patients with type 2 diabetes

2. Healthy kidney donors as controls
Standard donors based on the fundamental indication for kidney transplantation.
-age: 20-70 years
-Blood pressure(BP) <140/90 mmHg
-BMI<30
-Glomerular filtration rate (such as CCr) ≥ 80 ml/min/1.73m2
-UACR<30mg/gCre / UAE<150mg/day
-No diabetes
-No systemic disease (e.g. Malignancy, UTI, nephrotic syndrome, PKD, systemic TB, HIV, Creutzfeldt-Jakob disease)
Marginal donors
-age≤80
-BP<140/90 mmHg without anti-hypertensive drug or BP<130/80mmHg with antihypertensive drug + UACR<30mg/gCr
-BMI<32
-Glomerular filtration rate (such as CCr) ≥ 70 ml/min/1.73m2
-If diabetes, HbA1c<6.5% without insulin treatment & UACR<30mg/gCr

More detailed characteristics are summarized in Supplementary Table 1.

Recruitment

1. Renal biopsy cohort 1 (Protocol biopsy group)
Patients who agreed to join the NExT-DN Study (UMIN000024530) from 2/21/2017 to 12/1/2021

2. Renal biopsy cohort 2 (Clinical biopsy group)
I. Patients who underwent renal biopsy from 12/1/2010 to 2/21/2017 and were diagnosed with kidney disease.
II. Patients who were enrolled into previous clinical studies at Okayama University Hospital, Japan (No: 1009 and No: 1509-005[2063]).

3. Healthy kidney control who meet all of the following criteria
I. Donors of kidney transplantation at Okayama University Hospital, Japan from 7/1/2017 to 12/1/2021
II. Donors without urine abnormalities such as hematuria and proteinuria.
III. Donors who are not diagnosed with any kidney disease in the results of 0 hour-renal biopsy.
IV. Donors who agree to keep their blood and urine sample in OKADAI BIOBANK (Biobank of Okayama university hospital).

Ethics oversight

The protocol was approved by the Institutional Review Board of the Ethics Committee of Okayama University Hospital and registered with the University Hospital Medical Information Network (UMIN) (identification number: UMIN000046398). Written informed consent was obtained from all patients prior to their inclusion in the clinical study.

Note that full information on the approval of the study protocol must also be provided in the manuscript.

Field-specific reporting

Please select the one below that is the best fit for your research. If you are not sure, read the appropriate sections before making your selection.

- [x] Life sciences
- [ ] Behavioural & social sciences
- [ ] Ecological, evolutionary & environmental sciences

For a reference copy of the document with all sections, see nature.com/documents/nr-reporting-summary-flat.pdf

Life sciences study design

All studies must disclose on these points even when the disclosure is negative.

Sample size	No statistical methods were used to predetermine the sample sizes. In each experiment, sample size were determined based on the same experiment with the same group number performed in the previous paper. Sample sizes were indicated in the legend of each Figure and Supplementary Figure. For assays both in vitro and in vivo, there are generally at least 3-4 experimental/biological replicates for every assay and condition.
Data exclusions	No data were excluded from the analyses.
Replication	All attempts at replication were successful. Data reported in this manuscript were reproduced with at least 3 biologically independent replicates for the in vitro experiments and at least 3-4 independent mice per group.
Randomization	No randomization was performed for in vivo experiments, and experimental animals were analyzed based on age and sex-matched (male) controls. In other words, all male age-matched mice were used for observation and experiments. For the remaining in vitro experiments such as cell cultured experiments, all samples in one experiment were treated at the same time and with the same condition.
Blinding	Blinding was not performed in this study except for human IHC analyses since the investigators needed to know the genotype of animals and sample information for the downstream analyses. For histological analyses for human samples, clinical information was blinded for investigator/renal pathologists.

Reporting for specific materials, systems and methods

We require information from authors about some types of materials, experimental systems and methods used in many studies. Here, indicate whether each material, system or method listed is relevant to your study. If you are not sure if a list item applies to your research, read the appropriate section before selecting a response.

Materials & experimental systems

n/a	Involved in the study	
[ ]	[x]	Antibodies
[ ]	[x]	Eukaryotic cell lines
[x]	[ ]	Palaeontology and archaeology
[ ]	[x]	Animals and other organisms
[ ]	[x]	Clinical data
[x]	[ ]	Dual use research of concern
[x]	[ ]	Plants

Methods

n/a	Involved in the study	
[x]	[ ]	ChIP-seq
[ ]	[x]	Flow cytometry
[x]	[ ]	MRI-based neuroimaging

Antibodies

Antibodies used

Primary antibodies
Anti-GST Alexa Fluor 680 (Santa Cruz, sc-138 AF680) (1:2000)
Goat anti-Podocalyxin, biotinylated (R&D Systems, BAF1556) (2.5ug per mouse for podocyte isolation)
Guinea pig anti-Synaptopodin (Progen, GP94-N) (1:500)
Mouse anti-b-Actin (Cell Signaling, 4967) (1:5000)
Mouse anti–FLAG M2 monoclonal (Sigma-Aldrich, F3165) (1:500)
Mouse anti-STOML2 (Proteintech, 60052-1-lg) (1:1000)
Mouse anti-VDAC (Abcam, ab14734) (1:2000)
Mouse OXPHOS cocktail (Thermo Fisher, 45-8099) (1:500)
Rabbit anti-ATAD3A/B (Proteintech, 16610-1-AP) (1:1000)
Rabbit anti-a-Tubulin (Cell Signaling, 2144) (1:1000)
Rabbit anti-Calnexin (Proteintech, 10427-2-AP) (1:1000)
Rabbit anti-Catalase (Cell Signaling, 14097) (1:1000)
Rabbit anti-HA-Tag (Cell Signaling, 3724) (1:500)
Rabbit anti-Mitofilin (Proteintech, 10179-1-AP) (1:1000)

Rabbit anti-Ndufs4 (Novus, NBP1-31465) for all WB (1:500 for WB)
Rabbit anti-Ndufs4 (Abcam, ab137064) (1:100 for IHC of human paraffin section, 1:1000 for STED/STORM)
Rabbit anti-OPA1 (BD Biosciences, 612606) (1:1000)
Rabbit anti-PAX8 (Proteintech, 10336-1-AP) (1:5000)
Rabbit anti-Podocin (Sigma-Aldrich, P0372) (1:1000)
Rabbit anti-STOML2 (Proteintech, 10348-1-AP) (1:2000)
Rabbit anti-VDAC (Cell Signaling, 4661) (1:2000)
Rabbit anti-Wilms Tumor Protein (Abcam, ab89901) (1:1000)
Sheep anti-KIRREL3, biotinylated (R&D Systems, BAF4910) (2.5ug per mouse for podocyte isolation)

Secondary antibodies
Donkey anti–mouse Alexa Fluor 488 (Thermo Fisher, A21202) (1:2000)
Donkey anti–mouse Alexa Fluor 594 (Thermo Fisher, A21203) (1:2000)
Donkey anti–rabbit Alexa Fluor 488 (Thermo Fisher, A21206) (1:2000)
Donkey anti–rabbit Alexa Fluor 594 (Thermo Fisher, A21207) (1:2000)
Donkey anti–rabbit Alexa Fluor 647 (Thermo Fisher, A21244) (1:2000)
Goat anti-guinea pig Alexa Fluor 594 (Thermo Fisher, A11076) (1:2000)
Goat anti–mouse DyLight 680 (Thermo Fisher, 35519) (1:5000)
Goat anti–mouse DyLight 800 (Thermo Fisher, SA510172) (1:5000)
Goat anti-mouse ATTO 488 (Rockland, 610-152-121) (1:2000)
Goat anti–rabbit DyLight 680 (Thermo Fisher, 35519) (1:5000)
Goat anti–rabbit DyLight 800 (Thermo Fisher, SA535571) (1:5000)
Goat anti–rabbit IgG HRP Polymer (Vector Laboratories, MP-7451) (No dilution)

Validation

Primary antibodies
Anti-GST Alexa Fluor 680 (Santa Cruz, sc-138 AF680): https://www.scbt.com/p/gst-antibody-b-14
Goat anti-Podocalyxin, biotinylated (R&D Systems, BAF1556): https://www.rndsystems.com/products/mouse-podocalyxin-biotinylated-antibody_baf1556
Guinea pig anti-Synaptopodin (Progen, GP94-N): https://us.progen.com//anti-Synaptopodin-SYNPO-N-terminus-guinea-pig-polyclonal-serum/GP94-N
Mouse anti-Actin (Cell Signaling, 4967): https://www.cellsignal.com/products/primary-antibodies/b-actin-antibody/4967
Mouse anti–FLAG M2 monoclonal (Sigma-Aldrich, F3165): https://www.sigmaaldrich.com/US/en/product/sigma/f3165
Mouse anti-STOML2 (Proteintech, 60052-1-lg): https://www.ptglab.com/products/STOML2-Antibody-60052-1-Ig.htm
Mouse anti-VDAC (Abcam, ab14734): https://www.abcam.com/products/primary-antibodies/vdac1porin--vdac3-antibody-20b12af2-ab14734.html
Mouse OXPHOS cocktail (Thermo Fisher, 45-8099): https://www.thermofisher.com/antibody/product/OxPhos-Rodent-WB-Antibody-clone-Cocktail-Cocktail/45-8099
Rabbit anti-a-Tubulin (Cell Signaling, 2144): https://www.cellsignal.com/products/primary-antibodies/a-tubulin-antibody/2144
Rabbit anti-ATAD3A/B (Proteintech, 16610-1-AP): https://www.ptglab.com/products/ATAD3B-Antibody-16610-1-AP.htm
Rabbit anti-Calnexin (Proteintech, 10427-2-AP): https://www.ptglab.co.jp/products/CANX-Antibody-10427-2-AP.htm
Rabbit anti-Catalase (Cell Signaling, 14097): https://www.cellsignal.com/products/primary-antibodies/catalase-d5n7v-rabbit-mab/14097?country=JP&language=en
Rabbit anti-HA-Tag (Cell Signaling, 3724): https://www.cellsignal.com/products/primary-antibodies/ha-tag-c29f4-rabbit-mab/3724
Rabbit anti-Mitofilin (Proteintech, 10179-1-AP): https://www.ptglab.com/products/IMMT-Antibody-10179-1-AP.htm
Rabbit anti-Ndufs4 (Novus, NBP1-31465): https://www.novusbio.com/products/ndufs4-antibody_nbp1-31465
Rabbit anti-Ndufs4 (Abcam, ab137064): https://www.abcam.com/products/primary-antibodies/ndufs4-antibody-ep7832-ab137064.html
Rabbit anti-OPA1 (BD Biosciences, 612606): https://www.bdbiosciences.com/en-us/products/reagents/microscopy-imaging-reagents/immunofluorescence-reagents/purified-mouse-anti-opa1.612606
Rabbit anti-PAX8 (Proteintech, 10336-1-AP): https://www.ptglab.com/products/PAX8-Antibody-10336-1-AP.htm
Rabbit anti-Podocin (Sigma-Aldrich, P0372): https://www.sigmaaldrich.com/US/en/product/sigma/p0372
Rabbit anti-STOML2 (Proteintech, 10348-1-AP): https://www.ptglab.com/products/STOML2-Antibody-10348-1-AP.htm
Rabbit anti-VDAC (Cell Signaling, 4661): https://www.cellsignal.com/products/primary-antibodies/vdac-d73d12-rabbit-mab/4661
Rabbit anti-Wilms Tumor Protein (Abcam, ab89901): https://www.abcam.com/products/primary-antibodies/wilms-tumor-protein-antibody-can-r9ihc-56-2-ab89901.html
Sheep anti-KIRREL3, biotinylated (R&D Systems, BAF4910): https://www.rndsystems.com/products/human-mouse-rat-kirrel3-neph2-biotinylated-antibody_baf4910

Secondary antibodies
Donkey anti–mouse Alexa Fluor 488 (Thermo Fisher, A21202): https://www.thermofisher.com/antibody/product/Donkey-anti-Mouse-IgG-H-L-Highly-Cross-Adsorbed-Secondary-Antibody-Polyclonal/A-21202
Donkey anti–mouse Alexa Fluor 594 (Thermo Fisher, A21203): https://www.thermofisher.com/antibody/product/Donkey-anti-Mouse-IgG-H-L-Highly-Cross-Adsorbed-Secondary-Antibody-Polyclonal/A-21203
Donkey anti–rabbit Alexa Fluor 488 (Thermo Fisher, A21206): https://www.thermofisher.com/antibody/product/Donkey-anti-Rabbit-IgG-H-L-Highly-Cross-Adsorbed-Secondary-Antibody-Polyclonal/A-21206
Donkey anti–rabbit Alexa Fluor 594 (Thermo Fisher, A21207): https://www.thermofisher.com/antibody/product/Donkey-anti-Rabbit-IgG-H-L-Highly-Cross-Adsorbed-Secondary-Antibody-Polyclonal/A-21207
Donkey anti–rabbit Alexa Fluor 647 (Thermo Fisher, A21244): https://www.thermofisher.com/antibody/product/Goat-anti-Rabbit-

	IgG-H-L-Cross-Adsorbed-Secondary-Antibody-Polyclonal/A-21244<br>Goat anti-guinea pig Alexa Fluor 594 (Thermo Fisher, A11076): https://www.thermofisher.com/antibody/product/Goat-anti-Guinea-Pig-IgG-H-L-Highly-Cross-Adsorbed-Secondary-Antibody-Polyclonal/A-11076<br>Goat anti–mouse DyLight 680 (Thermo Fisher, 35519): https://www.thermofisher.com/antibody/product/Goat-anti-Mouse-IgG-H-L-Cross-Adsorbed-Secondary-Antibody-Polyclonal/35519<br>Goat anti–mouse DyLight 800 (Thermo Fisher, SA510172): https://www.thermofisher.com/antibody/product/Donkey-anti-Mouse-IgG-H-L-Cross-Adsorbed-Secondary-Antibody-Polyclonal/SA5-10172<br>Goat anti-mouse ATTO 488 (Rockland, 610-152-121): https://www.rockland.com/categories/secondary-antibodies/mouse-igg-hl-antibody-atto-488-conjugated-pre-adsorbed-610-152-121/<br>Goat anti–rabbit DyLight 680 (Thermo Fisher, 35568): https://www.thermofisher.com/antibody/product/Goat-anti-Rabbit-IgG-H-L-Secondary-Antibody-Polyclonal/35568<br>Goat anti–rabbit DyLight 800 (Thermo Fisher, SA535571): https://www.thermofisher.com/antibody/product/Goat-anti-Rabbit-IgG-H-L-Secondary-Antibody-Polyclonal/SA5-35571<br>Goat anti–rabbit IgG HRP Polymer (Vector Laboratories, MP-7451): https://vectorlabs.com/products/enzyme-polymer/immpress-hrp-goat-anti-rabbit-igg-kit

Eukaryotic cell lines

Policy information about cell lines and Sex and Gender in Research

Cell line source(s)	Conditionally immortalized mouse podocytes were a kind gift from Jochen Reiser (Rush University, Chicago, IL). HEK293T cells was purchased from ATCC.
Authentication	Immortalized mouse podocytes are regularly checked for podocyte like morphology and podocyte marker protein expression such as synaptopodin and podocin by Western blot. Knockdown or overexpression of the target gene was analyzed by qPCR and Western blot analysis. HEK293T cells were authenticated by manufacturer (ATCC) and confirmed by microscopy looking at morphology.
Mycoplasma contamination	All cell lines tested negative for Mycoplasma contamination.
Commonly misidentified lines (See ICLAC register)	No misdentified cell lines were used in this study.

Animals and other research organisms

Policy information about studies involving animals; ARRIVE guidelines recommended for reporting animal research, and Sex and Gender in Research

Laboratory animals	All mice were maintained in a temperature-controlled environment (22C) and 50-60% humidity under a 12-hrs light/dark cycle with free access to chow and water. Type 1 and type 2 diabetic mice (Ins2Akita/+ on C57BL/6J background and Leprdb/db on C57BLKS/J background) were obtained from Jackson Laboratories (Stock Nos. 003548 and 000642).
Wild animals	No wild animals were used in this study.
Reporting on sex	Only male mice were used in this study.
Field-collected samples	This study did not involve samples collected from the field.
Ethics oversight	All animal studies were reviewed and approved by the Institutional Animal Care and Use Committee of the University of Texas at MD Anderson Cancer Center and conducted according to the institutional and the US National Institutes of Health guidelines.

Note that full information on the approval of the study protocol must also be provided in the manuscript.

Clinical data

Policy information about clinical studies
All manuscripts should comply with the ICMJEguidelines for publication of clinical research and a completedCONSORT checklist must be included with all submissions.

Clinical trial registration	UMIN000046398 (UMIN: University Hospital Medical Information Network)
Study protocol	Study type: Retrospective cohort study (using previous data from ongoing prospective cohort study and retrospective cohort study)<br><br>Narrative objectives<br>To investigate novel targets and biomarkers to determinate pathophysiology and prognosis of diabetic kidney disease (DKD) using human renal tissue, blood, and urine.<br>Basic objectives<br>To explore the new therapeutic target of DKD<br><br>Eligibility<br>Age: not applicable<br>Gender: male and female

Key inclusion criteria
1. Renal biopsy cohort 1 (Protocol biopsy group)
Patients who agreed to join the NExT-DN Study (UMIN000024530) from 2/21/2017 to 12/1/2021
age: 20-80 years when obtaining informed consent.
Type 1 or type 2 diabetes
eGFR>15ml/min/1.73m2 at renal biopsy
2. Renal biopsy cohort 2 (Clinical biopsy group)
I. Patients who underwent renal biopsy from 12/1/2010 to 2/21/2017 and were diagnosed with kidney disease.
II. Patients who were enrolled into previous clinical studies at Okayama University Hospital, Japan (No: 1009 and No: 1509-005 [2063]).
3. Healthy kidney control who meet all of the following criteria
I. Donors of kidney transplantation at Okayama University Hospital, Japan from 7/1/2017 to 12/1/2021
II. Donors without urine abnormalities such as hematuria and proteinuria.
III. Donors who are not diagnosed with any kidney disease in the results of 0 hour-renal biopsy.
IV. Donors who agree to keep their blood and urine sample in OKADAI BIOBANK (Biobank of Okayama university hospital).

Key exclusion criteria
1. Renal biopsy cohort 1
People who decline the entry of NExT-DN Study (UMIN000024530).
2. Renal biopsy cohort 2
Patients who withdraw the agreement of previous clinical studies of our department (No: 1009 and No: 1509-005[2063]).
3. Healthy kidney control
People who decline the entry of this study.

Data collection

Clinical parameters at the time of renal biopsy and during follow-up periods after biopsy.
Histopathological parameters that is assessed by at least two nephrologists and renal pathologist.

Outcomes

Primary outcome
Correlation of novel biomarkers and pathophysiological targets in renal tissue, urine, and blood with pathological finding of diabetic kidney disease (DKD)
Secondary outcome
1. Investigation of clinicopathologic indicators of renal progression in DKD
I. eGFR 30-40% decline from baseline
II. eGFR decline > 3-5 ml/min/1.73m2/year
III. Commencement of dialysis or renal transplantation because of end stage renal disease
IV. Development of albuminuric stage

2. Investigation of other major complications of diabetes
I.Development of diabetic retinopathy
II.CVD event
III.Death

Flow Cytometry

Plots

Confirm that:

- [x] The axis labels state the marker and fluorochrome used (e.g. CD4-FITC).
- [x] The axis scales are clearly visible. Include numbers along axes only for bottom left plot of group (a 'group' is an analysis of identical markers).
- [ ] All plots are contour plots with outliers or pseudocolor plots.
- [x] A numerical value for number of cells or percentage (with statistics) is provided.

Methodology

Sample preparation	Podocytes were incubated at 37°C with fresh media without serum containing 5µM MitoSOX Red mitochondrial superoxide indicator (Thermo Fisher). The cells were analyzed by flow cytometry with help from UT-MDACC FCCICF Core.
Instrument	Beckman Coulter Gallios Flow Cytometer (Beckman Coulter)
Software	Kaluza Analysis Software (Beckman Coulter)
Cell population abundance	In total, 10,000 events were recorded for each sample and analyzed using Kaluza Analysis software.

Gating strategy	Live cells were defined based on forward and side scatter, single cells were defined on the basis of forward scatter area vs forward scatter height. Mitochondrial superoxide levels were assessed by measuring MitoSox Red MFI (median fluorescence intensity)

☐ Tick this box to confirm that a figure exemplifying the gating strategy is provided in the Supplementary Information.
